# Digital health and primary care: Past, pandemic and prospects

**DOI:** 10.7189/jogh.11.01005

**Published:** 2021-07-02

**Authors:** Claudia Pagliari

**Affiliations:** Global eHealth Group, Usher Institute, Edinburgh Global Health Academy & Centre for Population Health Sciences, The University of Edinburgh Medical School, Edinburgh, UK

## Abstract

This article reflects on the breadth of digital developments seen in primary care over time, as well as the rapid and significant changes prompted by the COVID-19 crisis. Recent research and experience have shone further light on factors influencing the implementation and usefulness of these approaches, as well as unresolved challenges and unintended consequences. These are considered in relation to not only digital technology and infrastructure, but also wider aspects of health systems, the nature of primary care work and culture, patient characteristics and inequalities, and ethical issues around data privacy, inclusion, empowerment, empathy and trust. Implications for the future direction and sustainability of these approaches are discussed, taking account of novel paradigms, such as artificial intelligence, and the growing capture of primary care data for secondary uses. Decision makers are encouraged to think holistically about where value is most likely to be added, or risks being taken away, when judging which innovations to carry forward. It concludes that, while responding to this public health emergency has created something of a digital ‘big bang’ for primary care, an incremental, adaptive, patient-centered strategy, focused on augmenting rather than replacing existing services, is likely to prove most fruitful in the longer term.

## PRIMARY CARE AND THE SHOCK OF THE PANDEMIC

What exactly constitutes ‘primary care’ is still somewhat contested, with definitions of the concept ranging from the philosophical and aspirational to the concrete and practical [1], while its professional scope varies across health systems [2]. For the purposes of this article, the term is used synonymously with family medicine or general practice, as described by the World Health Organization Europe [3].

The primary care setting is typically the first point of contact for patients seeking medical treatment, the setting in which most clinical and chronic disease management services are delivered, and a locus of referral to specialist care. It covers a wide variety of health needs, types of patients and processes, and often involves multidisciplinary teams. Patients and their families tend to have long-term relationships with their primary care doctor or practice, who typically holds their lifelong health record. Primary care may also integrate with other services, such as when delivering public health screening programs or managing patients with complex conditions in collaboration with clinical specialties. Making the best use of digital health requires careful consideration of these complexities, as well as technologies themselves.

Media headlines in the first wave of the coronavirus crisis were dominated by pressures on hospitals, but as the frontline of medicine in many countries, primary care services were also severely disrupted. Despite the initial shock, most were quick to adapt, due in large part, to the use of digital technologies, but this change has been easier and more rapid in some health systems than in others.

## BUILDING ON THE STATUS QUO

Many digital health innovations have been available in primary care settings for some time, although the extent to which they are used still varies widely, both between and within nations.

Electronic health records are a mainstay of primary care in many countries, integrating structured and unstructured patient data alongside coding systems, clinical decision support, e-prescribing, e-referral, and other features. Use of these systems is still patchy, however. While in some European countries, such as the UK and the Netherlands, primary care has been ‘paperless’ for years, elsewhere pockets of resistance can be seen [4]. Often this has less to do with money than professional cultures, silo-working, the absence of a strategy for primary care computing, or multiple public/private sector models that have hindered standardization. The coronavirus pandemic has shone further light on these systemic challenges and emphasized the value of strong information systems for safe, accurate, effective and coordinated care [5].

Hybrid digital methods, designed to support rather than replicate in-person care, are also being widely used. These include SMS appointment reminders (text messages), which can help to reduce ‘no shows’, and check-in kiosks which reduce administration time in primary care offices [6]. During COVID-19, mobile phone check-in may help to minimize the time spent in shared waiting rooms [7], increasing both efficiency and patient safety, given the risks of physical and airborne transmission [8].

Clinical queries sent via email or secure messaging systems can be useful for primary care patients seeking written health advice and clarification of medication or treatment plans, and may help to quickly differentiate the worried well from those requiring physical assessment. Wide international variation in the use of email consultation has been reported, however, and in Europe overall levels of usage remain low, with little indication that this has changed during the pandemic [9]. Concerns about workload, legal liability, confidentiality and patient safety have often been cited as barriers to implementation, although there is scant evidence of negative effects [10]. Policies aimed at ensuring that doctors can be reimbursed for email consultations have been introduced in some countries, but have had mixed success in changing practice [11] and new legal requirements, such as those in Denmark [12], may be needed.

Online patient portals and related apps are being used in primary care for appointment booking, ordering repeat medications and viewing test results, but access to and use of more detailed personal health records has lagged behind, even in countries where patients, in theory, have rights of access [13]. Opportunities for patients to view their medical notes and contribute personal health observations or device readings could improve empowerment and collaborative care for those undergoing treatment [14]. Health professionals have been somewhat reluctant to implement this functionality, however, largely due to concerns over added workload [15].

Although a number of countries, such as Germany [16], have brought patient portals onstream during the pandemic, most were already planned. Patient portals can also offer other potentially useful features, such as the ability to download relevant documentation when required for work, insurance or travel, which may have particular advantages in the context of social distancing. For example, England’s NHS App now features a vaccine certification module, with print capabilities, which is likely to encourage many more citizens to adopt this portal technology in the coming months [17].

Experience of remote, virtual or tele-consulting has been growing over the last 20 years, and while fears over losing the human touch have dampened enthusiasm in primary care, trials have shown it to be acceptable and useful for some patients [18]. The pandemic has provided the catalyst needed to propel this into routine practice and there is huge optimism about its ongoing use. Recent experience at scale has yielded several lessons, however. On the one hand, adaptation to remote consulting has been easier than expected, helping to alter professional and patient perceptions. On the other hand, videoconferencing has proven less necessary or desirable than had previously been assumed, with telephone calls being equally or more acceptable in most cases [19]. Moreover, while video consulting has advantages for ‘eyeballing’ physical cues [20], it may also substantially increase general practitioner workload compared with telephone consulting [21]. As such, the rush of new video platform start-ups into the global telehealth market may be creating unnecessary demands and costs, which call for more guidance and standards [22]. Government-funded video consulting platforms are being rolled out in some countries but their normalization and sustainability in practice remains to be seen [23]. More ‘frugal’ forms of remote telemonitoring also hold potential, such as using SMS to enable ‘automated hovering’ over patients managing coronavirus symptoms at home, in a way that can trigger human intervention if the need arises [24].

Other forms of primary care telehealth have already moved into routine use, with clinical guidelines now recommending self-management using well-calibrated home monitoring devices, alongside usual care, for patients with long-term conditions [25]. During the pandemic the use of such devices is reported to have increased dramatically, although this has also raised concerns over maintaining their robustness, accuracy, privacy and safety [26].

## EMERGING INNOVATIONS

Looking to the future, we can increasingly expect to see first-line queries triaged by chatbots, with or without Artificial Intelligence, which may direct patients to self-care advice or refer them to a clinician, depending on the answers to successive questions [27]. As the accuracy and safety of these tools are still in question, however, the risk threshold for triggering human intervention needs to be fairly low, thus introducing such technologies may potentially add, rather than reduce clinical time pressures at this stage [28].

Wearables create similar dilemmas. On the one hand, they provide more ambulatory data and can give more freedom to the patient. On the other hand, they can pose greater responsibility for clinicians to monitor this data, if used as part of a supported care plan. Continuous monitoring also has potential to trigger hospital admission through false positives or simply by surfacing symptom readings that would not have been apparent before, as has been seen in patients with COPD [29,30]. While certain wearables are already being deployed successfully in primary care, for example in ambulatory blood pressure monitoring, as the use of such devices becomes more common and diverse, so will be the volume, variety and complexity of the readings they generate. This is likely to become problematic for clinicians to manage and interpret, calling for the introduction of user-friendly and clinically accurate multi-parameter dashboards, as well as further standards to ensure safety and cost-effectiveness [31].

Wearables have nonetheless proven useful for enabling care at a distance during the pandemic, such as for monitoring vital signs in patients recuperating at home, where usual community-based follow up has been curtailed [32].

The integration of machine learning and Artificial Intelligence into clinical software, decision support tools and apps in primary care has potential to help optimize and personalize treatments. However, it will be essential for clinicians to recognize the risk of bias and harm when using these technologies, and ensure that they have been appropriately tested, regulated and approved. They also need to recognize that algorithms, even if technically robust, may be fed by data or evidence which is flawed, incomplete, unrepresentative or outdated. As such, despite the promise of such methods, the continued need for sound clinical judgment cannot be understated [33].

Introducing speech recognition technology into the consulting room has been proposed as one way of reducing administrative burden, by automating clinical documentation. While the use of voice assistants as ‘digital scribes’ has been trialled, technical, structural, ethical and legal difficulties mean these are still some way from routine use [34].

Other digital innovations likely to impact primary care practice include diagnostic ‘laboratory-on-a-chip’ devices, for analysing biosamples, which have potential to improve efficiency by quickly surfacing clinical insights previously only measurable in specialist facilities [35]. Similarly, with more sophisticated imaging capabilities being integrated into smartphones, we can anticipate greater use of pocket-sized visual diagnostics in the context of primary care and community medicine [36].

## WORK, ORGANIZATION AND VALUE

The structure of primary care work has also evolved over time, with single-handed practitioners increasingly joining collaborative partnerships and even multi-specialty primary care centres, in places where the population is large enough to sustain them [37]. Such centres may be able to offer a much wider range of services, including mental health and minor surgery, using technology-supported triage to direct patients to the most appropriate and cost-effective service for their health issue. While the latter approach may not be feasible in many settings, in a future pandemic it might be possible to rapidly assemble and deliver this virtually, with the right digital platforms and agreements. Some countries have already launched ambitious plans for national-scale digital triage, catalysed by COVID-19. This includes NHS England’s ‘digital first’, strategy, which aims to ensure that all primary care is routed through an online triaging system, which directs patients to either online, telephone, or video consulting before a face to face consultation [38].

Despite the attractions of ‘smarter’ service allocation, health service planners must be careful to avoid technocentric over-optimism, which neglects the therapeutic value of clinical relationships and care continuity. Having a known family doctor who cannot only access the patient’s digital record, but also has an informal knowledge of their health history, needs and circumstances, offers considerable advantages in primary care. For example, it may provide more context for symptom interpretation, empathy for understanding barriers to treatment compliance, and a trusted environment for sharing health concerns or debunking misinformation, as we have seen so much of during the coronavirus ‘infodemic’ [39]. While digital hand-off to any suitably qualified practitioner may be safe and acceptable for patients presenting with minor acute illness, for those with complex needs or the elderly, this is likely to be a false economy [40].

Wholesale conversion to online consulting in primary care is also ill-advised. Patients may have difficulties articulating their symptoms at a distance, and clinic-based follow up and testing will be needed to minimize the possibility of harm from undiagnosed disease, particularly in health systems where specialist medicine is accessed via primary care referrals. Likewise, failing to appropriately escalate patients from protocolized telephone advice to medical care may have serious clinical consequences and can damage trust in legitimate telehealth services, calling for high levels of vigilance and rigorous training for call handlers [41].

Experience of remote telemonitoring in primary care has also revealed the importance of initial training for patients and professionals, to gain the most value and avoid expensive or dangerous errors [42]. Managers should factor this into their cost-calculations, and not assume a plug-and-play scenario.

Pressures on clinicians to manage more patients without additional time, have been blamed for professional burnout, which has been magnified during the coronavirus crisis [43]. While some types of AI could help to alleviate administrative burdens or act as a cognitive prosthesis for clinicians making complex decisions at speed, reliance on ‘black box’ decision support could, paradoxically, exacerbate moral stress if clinicians are unable to judge the validity or safety of algorithmic recommendations [44]. As such, thinking carefully about how AI fits with or could disrupt clinical workflow and expertise, will be vital if these systems are to be safely introduced.

## THE ROLE OF HEALTH SYSTEMS

It is not possible to consider the future of digital primary care without also considering the broader health systems in which this is taking place. Although universal health coverage is regarded as the gold standard, many countries still rely on a combination of government and self-funded primary care, whether out-of-pocket or via private insurance [45]. Digital health may create opportunities for patients in these settings to manage their health more affordably, by encouraging preventive lifestyle changes, facilitating disease self-management, and by lowering the direct and indirect costs of clinic visits, travel or unpaid time off work. More research is needed to verify these savings, however, with most such predictions coming from the industry itself [46].

Even in countries with free access to primary care, such as the UK, new businesses offering to deliver private video consulting services and rapid appointments are proliferating. While these offer speed and convenience for patients with the money to pay, they also increase existing health inequalities and risk eroding the state-funded primary care workforce. It may also tempt some governments to nudge patients towards paying twice for their care - once through their taxes and once from their taxed salaries, creating unfairness even for those with the most resources.

Primary care is a luxury that people in many countries can’t easily obtain, either because there are too few doctors, or communities are too remote and widespread to sustain local health facilities. For this reason, the value proposition for remote or computer-driven primary care may be greater in resource-constrained regions. For example, using smartphone protocols to augment the skills of a community health worker may provide a ‘virtual’ nurse or doctor in human form [47], chatbots or simpler mobile phone question-answering tools may significantly improve preventive and self-care, and telehealth services may connect people in communities to primary care doctors located elsewhere [48].

Meanwhile some branches of the digital tech industry are exploring opportunities to by-pass conventional primary care altogether. For example, China’s Ping An Good Doctor has been rolling out unstaffed AI-assisted telemedical booths in shopping centres, which can undertake triage, preliminary diagnostics and even prescribing, while automatically referring the attendee for specialist care if merited [49]. While technologies like this may offer an affordable option for patients in highly unequal and underserved health systems, such impersonal approaches are likely to be resisted in Europe and other countries with strong primary care traditions.

As already noted, global health systems vary widely in their digital maturity, for historical reasons, such as the prioritization of technology and information infrastructures, policies and incentives favouring innovation, and workforce adaptability [5]. Likewise, countries’ ability to respond to public health emergencies like COVID-19 is also bound up with the broader organization and funding of their health systems, particularly differences in the provision of universal health coverage. Deficiencies in the US response, for example, have been blamed on under-resourced, disconnected, unequal and unaffordable primary care [50]. As other experts have noted, weak primary health care systems don’t suddenly become strong because you introduce digital health [51].

## DATA, CONTROL AND TRUST

The need for health data is magnified during public health emergencies and, as the holder of most citizens’ health records, primary care is one the richest sources of such data in many countries. During the COVID-19 pandemic, failures to adequately utilise primary care data to support a coordinated public health response have been sharply criticized [52]. Patient data from primary care have nevertheless been harnessed and linked with other sources of data, to inform national outbreak surveillance [53], understand service pressures [54], seed biomedical research [55], develop risk algorithms [56] and analyse inequalities to inform clinical and public health interventions [57,58], amongst other uses.

While professional obligations and regulations have tightly controlled the release of primary care records for secondary uses in the past, a number of countries are now beginning to automate the extraction of coded patient data from primary care information systems, at scale, into centralized (or functionally centralized) digital platforms [59]. The linkage and analysis of this ‘big data’ has potential to inform improvements in health care efficiency, quality, treatments, disease prevention and patient outcomes, and has led to some valuable insights about COVID-19 [60]. However, it also creates significant privacy and security risks, which have not yet been fully put to the test and, if mishandled, may damage public trust [61]. This trust can be particularly fragile when commercial actors are involved, as has been seen in debates over the role of global technology giants in the pandemic response [62]. Meaningful public consultation, appropriate anonymization, opt-out mechanisms, strong security and effective regulatory oversight will all be needed to ensure that these uses of patient information are acceptable, safe, proportionate, fair and legal. This is relevant to primary care practice, not only because of the type of personal data it generates, but also because many patients believe their trusted family doctor has control over decisions about how their health information may be re-used, which is increasingly no longer the case, creating room for tension in the clinical relationship. It may also disempower primary care doctors who have previously had greater control over their patients’ information, although this may be partially balanced through reciprocal data reports that can help to improve patient care [63]. Insensitive handling of such projects also risks significant legal push-back and reputational damage, as is currently being seen in the UK, fuelled in part by suspicions of data opportunism in the context of COVID-19 [64].

Aside from such major programs, which are often directed by governments, the data generated through the use of digital technologies in primary care is also being mediated by a plethora of businesses, such as providers of software, devices, apps and cloud hosting services, whose information governance processes can be hard to fathom [65]. As the use of digital tools in primary care grows, so will the need for clinicians and managers to ensure suppliers are adhering with relevant privacy and security requirements. Being able to rely on a trusted, standardized, verified and accountable digital health ecosystem will help to reduce this burden, but such collaborative enterprises also need effective governance, and not only by the industry itself [66].

These issues are being amplified as digital platform giants like Amazon, Apple and Google move into primary care. While most are currently prioritising telehealth and digital triage, their vast data ecosystems, coupled with advanced analytics capabilities, put them in a unique position to learn from and synthesise human decision-making, and potentially even empathy, raising new ethical dilemmas around patient trust and safety [67].

## UNDERSTANDING PATIENTS AND COMMUNITIES

Just as with primary care professionals and organizations, the patients and communities they serve can vary widely in their readiness for digital change. Patient characteristics, circumstances and preferences can also influence the value that may be obtained from different digital health opportunities.

Those who are already familiar with video consulting may benefit more from this medium than others, for example, while email consulting may offer welcome time-saving for young professionals but leave the elderly disempowered and under-treated [68]. Similarly, while access to detailed health records may greatly benefit some patients, it may be confusing or alarming for others, could be misused by coercive actors, or may unintentionally betray the confidentiality of third parties, calling for careful conversations with patients at risk, and appropriate data redaction efforts [69].

Long-standing social inequalities in health status, health literacy and health empowerment are also manifested in the ‘digital health divide’, whereby those with the means to access and use these new opportunities can further enhance their health, whilst those without may fall further behind. As the frontline of medicine, primary care needs to pay special attention to digital inclusion [70]. In practical terms, cheap and accessible approaches, like telephone, SMS text messaging and print may be preferable to sophisticated smartphone apps for reaching low income communities or the elderly, and in many cases are just as effective. As digital gradually becomes the default mode for accessing services or obtaining support between consultations, novel approaches such as ‘prescribing’ digital devices and training, or deploying digitally-skilled patient navigators, will be needed to ensure fair access for those with the greatest need [71].

Anecdotal reports in the early days of home telemonitoring suggested that some doctors were reluctant to trust the data being brought to them by patients, preferring to rely on episodic in-clinic measures, despite richer information being available from continuous recording. With more standards in place and wearables now being directly issued in primary care, this barrier is likely to reduce, although trust in patient-generated data remains highly conditional [72]. Recent studies of telehealth suggest that while patients with serious long-term conditions may be greatly comforted by having their symptoms monitored at a distance, some doctors fear that this will encourage dependency, illustrating how value judgements can also influence decision making [73].

So called Health 2.0 has impacted primary care since the dawn of the Internet, with greater access to online health information enabling patients to have more informed discussions with their doctors and doctors to direct their patients to relevant resources. Professional concerns about the clinical time needed to correct misunderstandings about evidence or treatments have previously been expressed [74]. The dramatic rise of social media misinformation and disinformation during the pandemic suggests that new training and skills in infodemic management will be needed if primary care is to remain practically manageable and maintain mutual trust and respect in the therapeutic encounter [75].

Understanding people’s personal circumstances and attitudes is not only vital for ensuring the appropriate use of alternative methods but can also be critical for their effective design. Previous waves of telehealth optimism have foundered on insufficient understanding of patient needs and primary care workflow, leading to poor design decisions, involving intrusive technologies or unrealistic expectations for clinician monitoring [76,77]. Accounts of patients left hanging around in simulated waiting rooms during the pandemic echo this failure and call for designing with and for users, not just digitizing existing services [78].

## MOVING FORWARD

As we move beyond the pandemic, new norms around digitally-augmented and collaborative medicine are likely to develop, reflecting both generational and technological trends, but human primary care will remain a vital requirement for effective, fair and sustainable health systems [79].

Unresolved challenges around privacy, patient safety, equity, rights, empathy and trust, call for more attention to be paid to the unique ethical challenges of digital health in primary care and new guidelines, tailored to this specific context, are warranted.

When the public health emergency ends, digital first approaches and expedited data sharing agreements that were implemented out of necessity should be reviewed. Evidence on the effectiveness of digital health in primary care is still nascent [80] and while it makes sense to harness the momentum of the pandemic to modernize aspects of the service, serious thought should be given as to what other valuable aspects of primary care may be lost in the process.

Digital transformation in primary care has occurred in incremental steps, but remarkable progress has been achieved over time. Experiences gained during the pandemic have proven useful for driving adoption, altering mindsets and learning lessons, but given the complexities involved in this human-centred discipline, further progress is likely to be gained through an ongoing process of evolution and adaptation rather than revolution and disruption.
